# Supplementary figures and images for: Reading canonical and modified nucleobases in 16S ribosomal RNA using nanopore native RNA sequencing
Source: PLoS One. 2019 May 16;14(5):e0216709. doi: 10.1371/journal.pone.0216709 (PMC6522004; doi:10.1371/journal.pone.0216709)

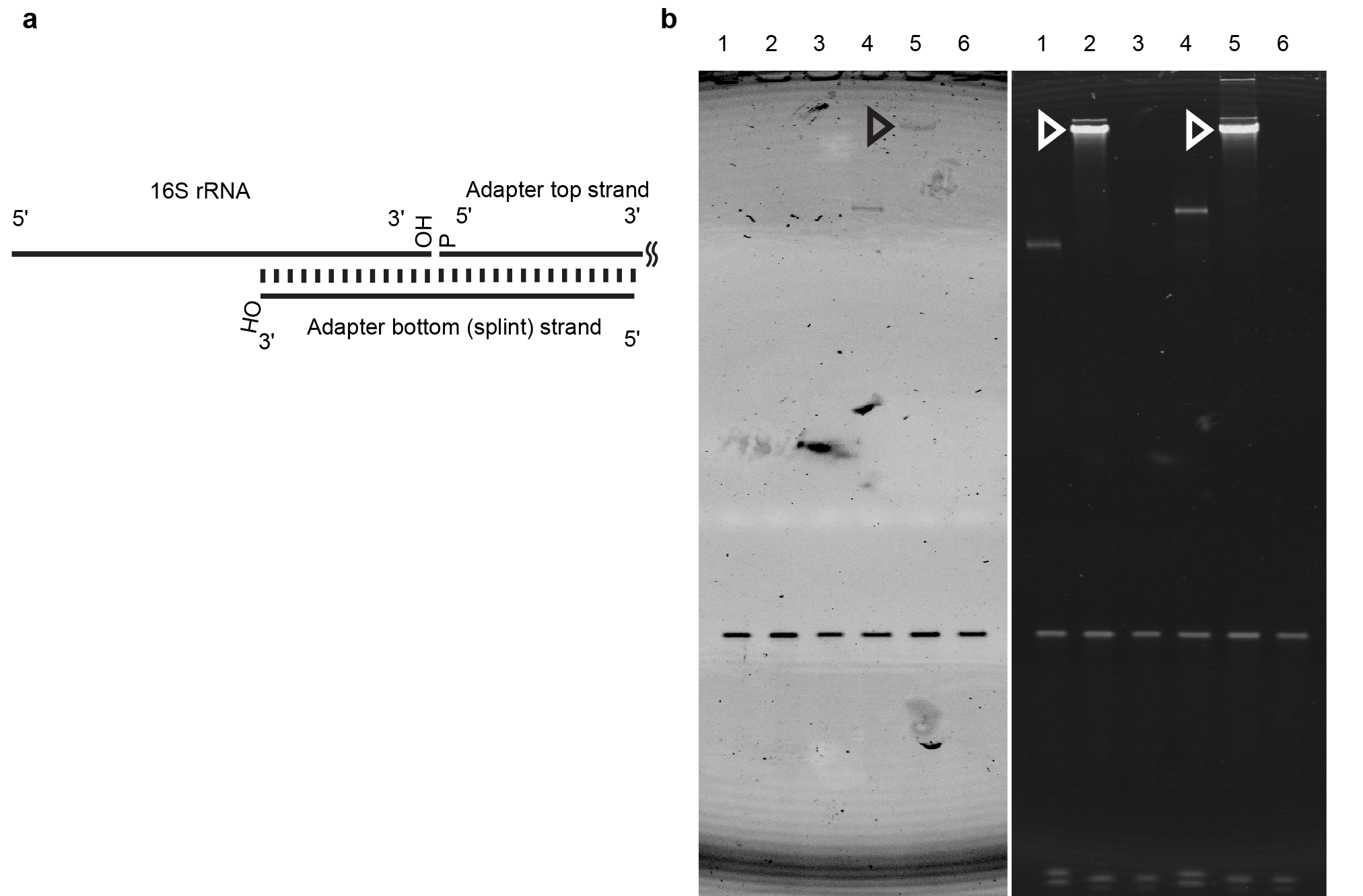

Supplement: S1 Fig — (a) Schematic of oligonucleotide adapter hybridized to the 3′ end of 16S rRNA. The adapter bottom (splint) strand can hybridize 20 nt to the conserved Shine-Dalgarno sequence on the 16S rRNA 3′ end. The adapter top strand can hybridize 20 nt to the 5′ end of the adapter bottom strand as shown. The 3′ terminal hydroxyl of the 16S rRNA and the 5′ terminal phosphate of the adapter top strand can be covalently joined by T4 DNA ligase. The broken line indicates an unshown ssDNA 3′ overhang, which is necessary for ligating the ONT sequencing adapter (see Methods). (b) Denaturing acrylamide gel analysis of a ligation reaction demonstrating the 16S adapter hybridizes and ligates to E. coli 16S rRNA 3′ ends. The left panel shows the unstained gel image. The lower band is a fluorescent, 3′-6-FAM-labeled version of adapter top strand. Lanes 1–3 show pre-ligation reaction samples for: Lane 1) RNA adapter ligation positive control with a polyA-specific adapter containing a 3′ terminal oligo dT10 overhang (replaces 16S-specific overhang) and the 6-FAM labeled top strand. A synthetic 288mer polyA RNA is used as the control substrate. Lane 2) 6-FAM-labeled 16S rRNA-specific adapter and purified 16S rRNA from E. coli. Lane 3) ligation negative control with adapter present, E. coli 16S rRNA, but without T4 DNA ligase. Lanes 4–6 show post-ligation reaction samples for: Lane 4) RNA adapter ligation positive control with polyA RNA 288mer. Lane 5) 16S rRNA reaction with 6-FAM labeled 16S rRNA-specific adapter. Lane 6) ligation negative control. The size-shifted fluorescent top strand indicates ligation to the 16S rRNA 3′ end (Open arrow). The right image is the same gel stained with SybrGold. Position of the 16S rRNA is indicated by open arrows. (PNG) [file pone.0216709.s001.png]

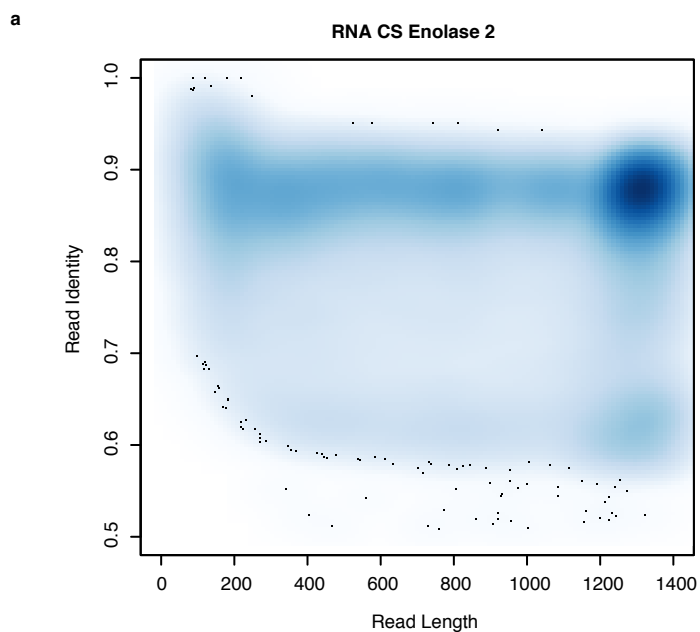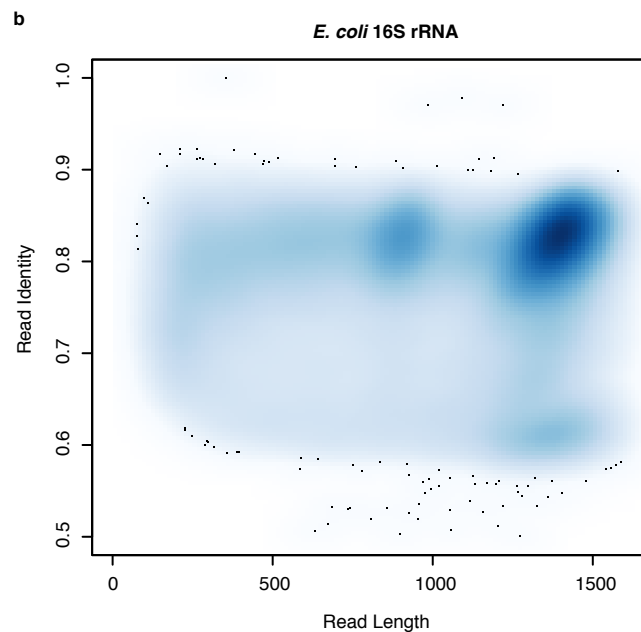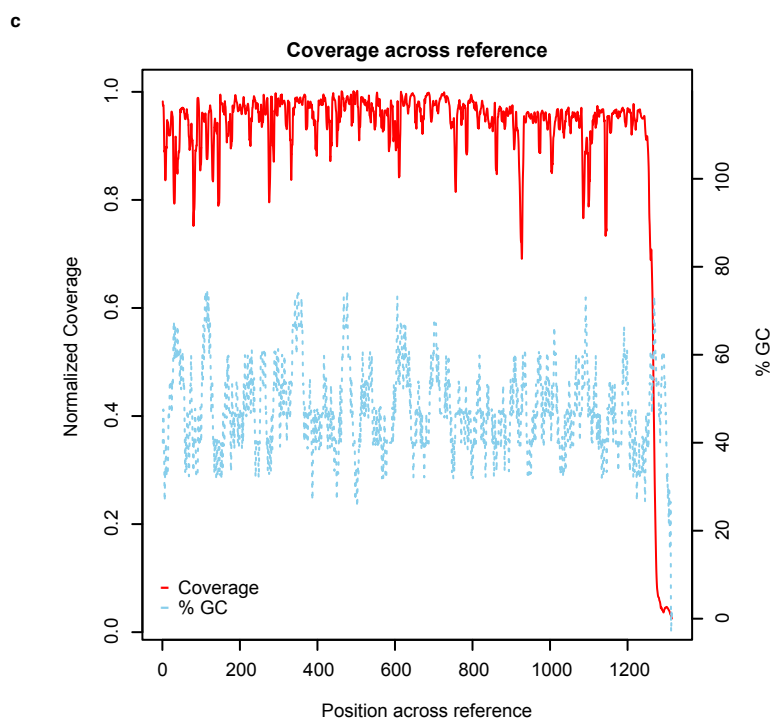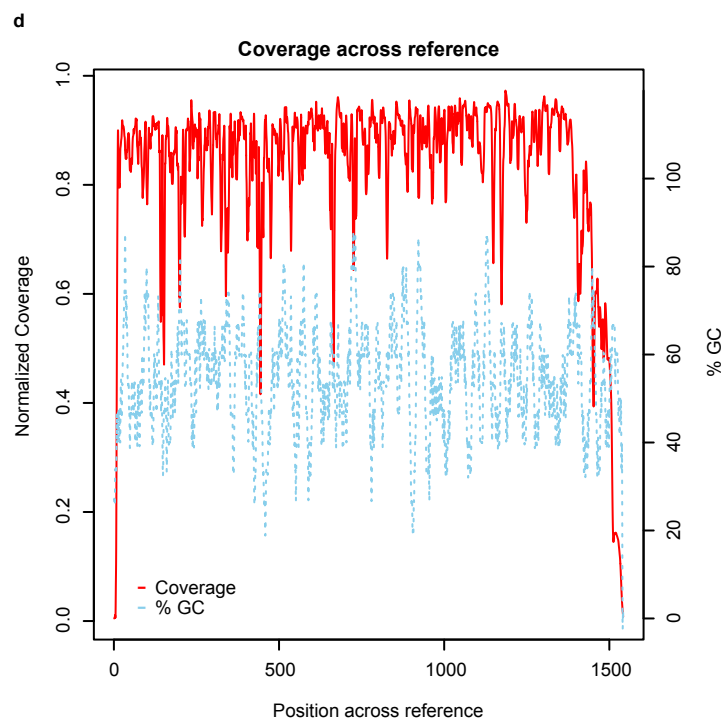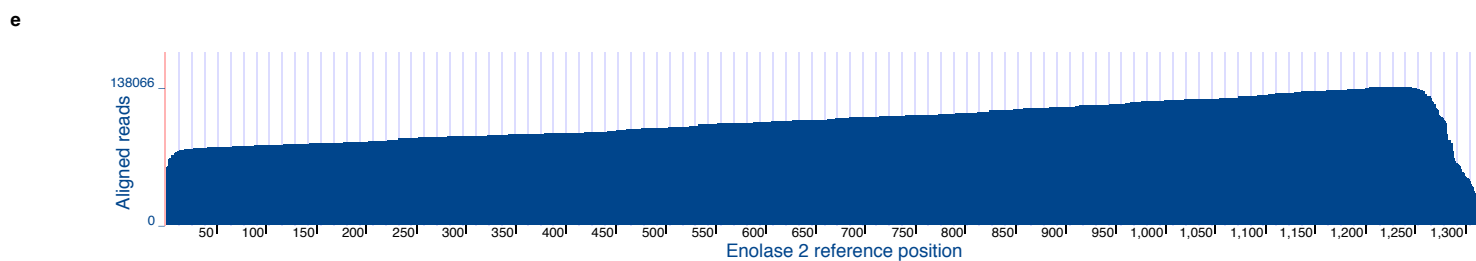

Supplement: S2 Fig — Alignments were performed using marginAlign (guide alignments from BWA MEM “-x ont2d” followed by chaining). (a) Identity vs. read length for Enolase 2. The intensity of blue shading represents the density of the data distribution. The black dots at the edge of the distributions represent regions where there are only a few data points. (b) Identity vs. read length for 16S E. coli rRNA. The blue shading and black dots are as in panel (a). (c) Coverage across reference for Enolase 2 calibration strand. (d) Coverage across reference for 16S E. coli rRNA. (e) Alignment of 100,000+ Enolase 2 reads to the reference sequence. (PDF) [file pone.0216709.s002.pdf]

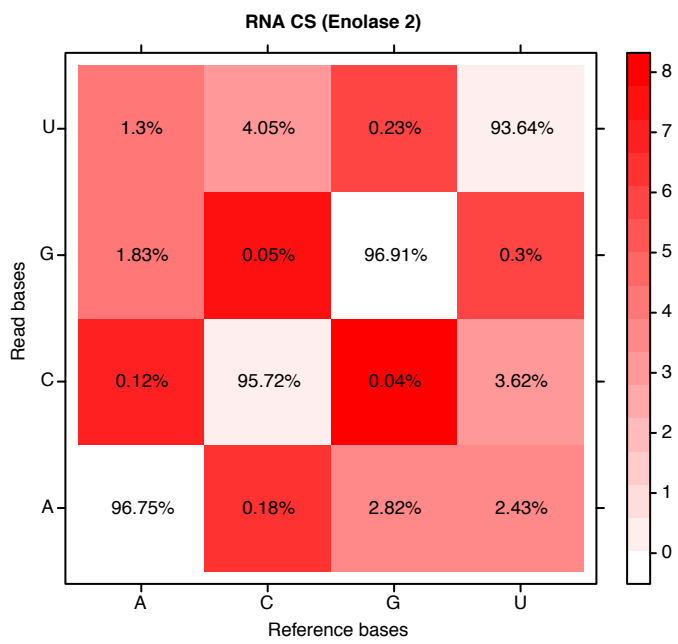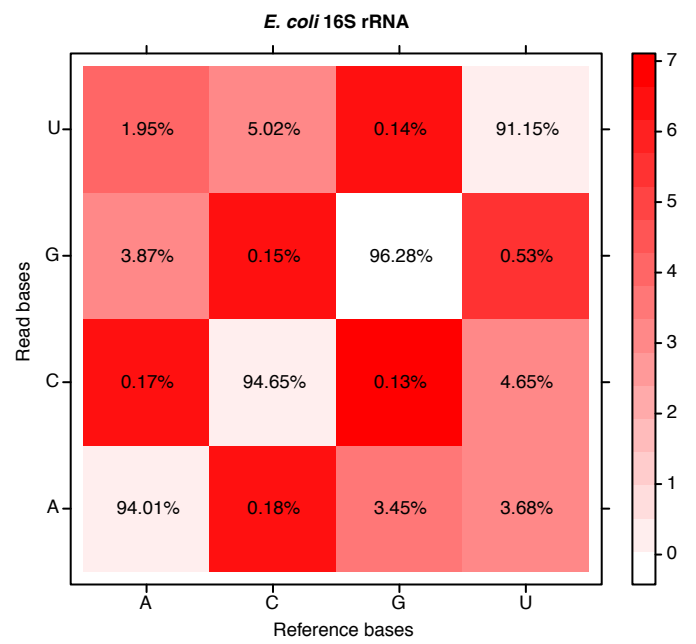

Supplement: S3 Fig — This matrix was determined using marginAlign EM. The matrix shows low rates of C-to-G and G-to-C substitutions, relative to the other substitutions. The color scheme is fitted on a log scale, and the substitution values are on an absolute scale. (PDF) [file pone.0216709.s003.pdf]

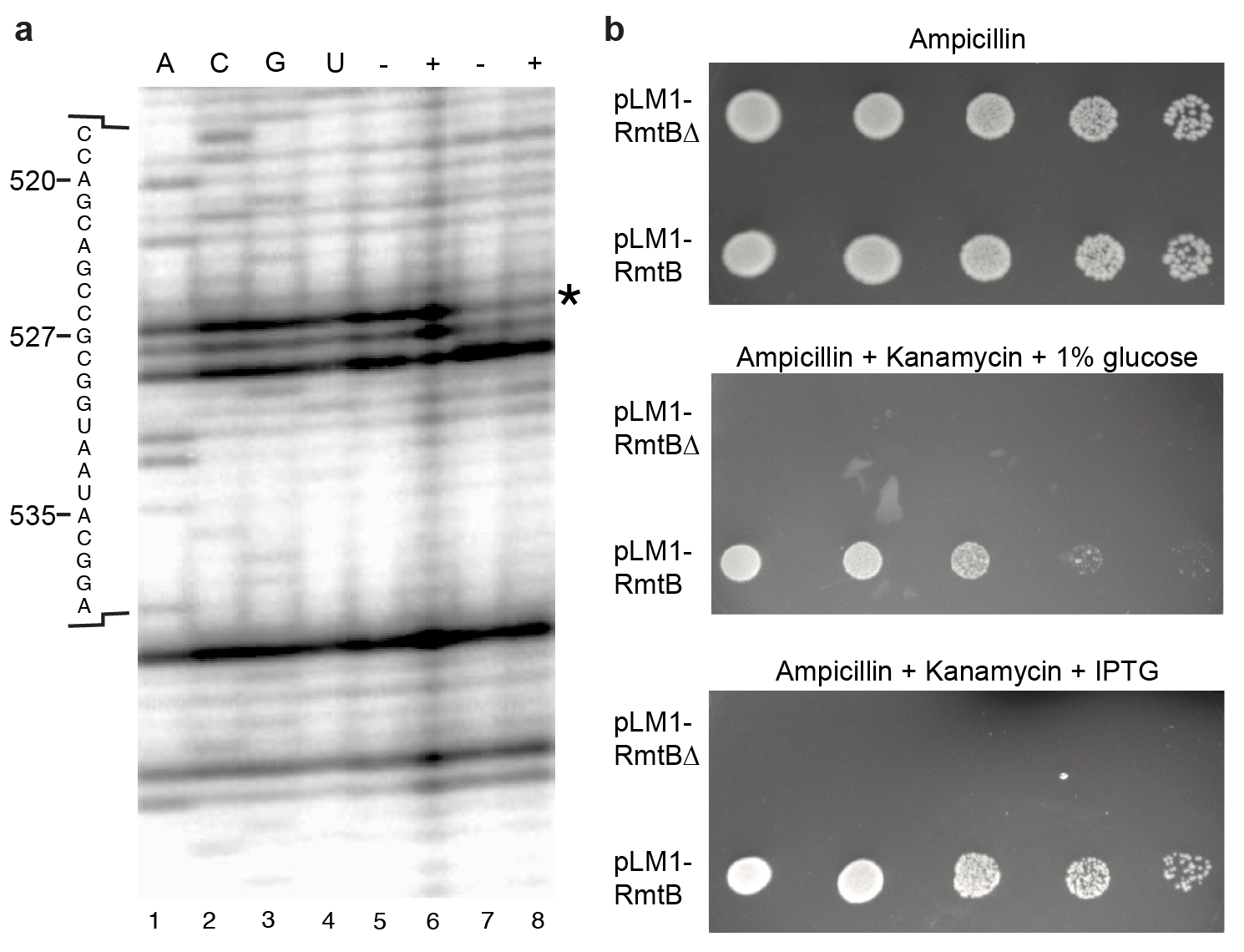

Supplement: S4 Fig — (a) Canonical m7G527 is present in wild-type E. coli and absent in the RsmG deficient E. coli strain. Sodium borohydride/aniline cleavage was used to assess the presence or absence of m7G527 in 16S rRNA from wild-type E. coli str. MRE600 or RsmG deficient (mutant) E. coli str. BW25113 JW3718Δ. Sequencing lanes 1–4 are labeled for A, C, G, and U of the RNA sequence. The respective lanes are reactions containing the complementary ddNTP to the RNA sequence. Wild-type 16S rRNA from E. coli str. MRE600 is used as the template. Lanes 6 and 8: sodium borohydride/aniline treatment (labeled +) of 16S rRNA from wild type E. coli and 16S rRNA from RsmG mutant E. coli, respectively. Strand cleavage, which is dependent on the presence of m7G, should result in an primer extension stop at C528, 1-nt preceding G527 (position 527 is marked by an asterisk). Lane 5 and 7: untreated 16S rRNA for wild type and mutant 16S rRNA. Primer extension products were run on denaturing 6% acrylamide gel, and imaged using a phosphorimager. A spontaneous RT stop appears at m7G527 in all lanes where wild-type 16S rRNA was used as the template, which has been observed previously. (b) RmtB confers a kanamycin resistance phenotype consistent with G1405 N7-methylation in 16S rRNA from an engineered E. coli strain. Serial dilutions from 10−2 to 10−6 (Left to Right) of E. coli BL21 DE3 pLysS strains transformed with pLM1-RmtB and negative control pLM1-RmtBΔ were spotted on LB agar plates. The pLM1 plasmids use pET32a as the backbone, which contains an ampicillin resistance gene. The RmtB gene is under the control of a lactose inducible T7 promoter. Plates are supplemented with: 100 μg/ml Ampicillin (top), 100 μg/ml Ampicillin + 200 μg/ml Kanamycin + 1% glucose (middle), 100 μg/ml Ampicillin + 200 μg/ml Kanamycin + 1 mM IPTG (bottom). Apparent leaky expression of the pLM-RmtB vector leads to some cell survival, even under non-inducing conditions and in the presence of glucose. RmtB is known to confer high-l [file pone.0216709.s004.png]

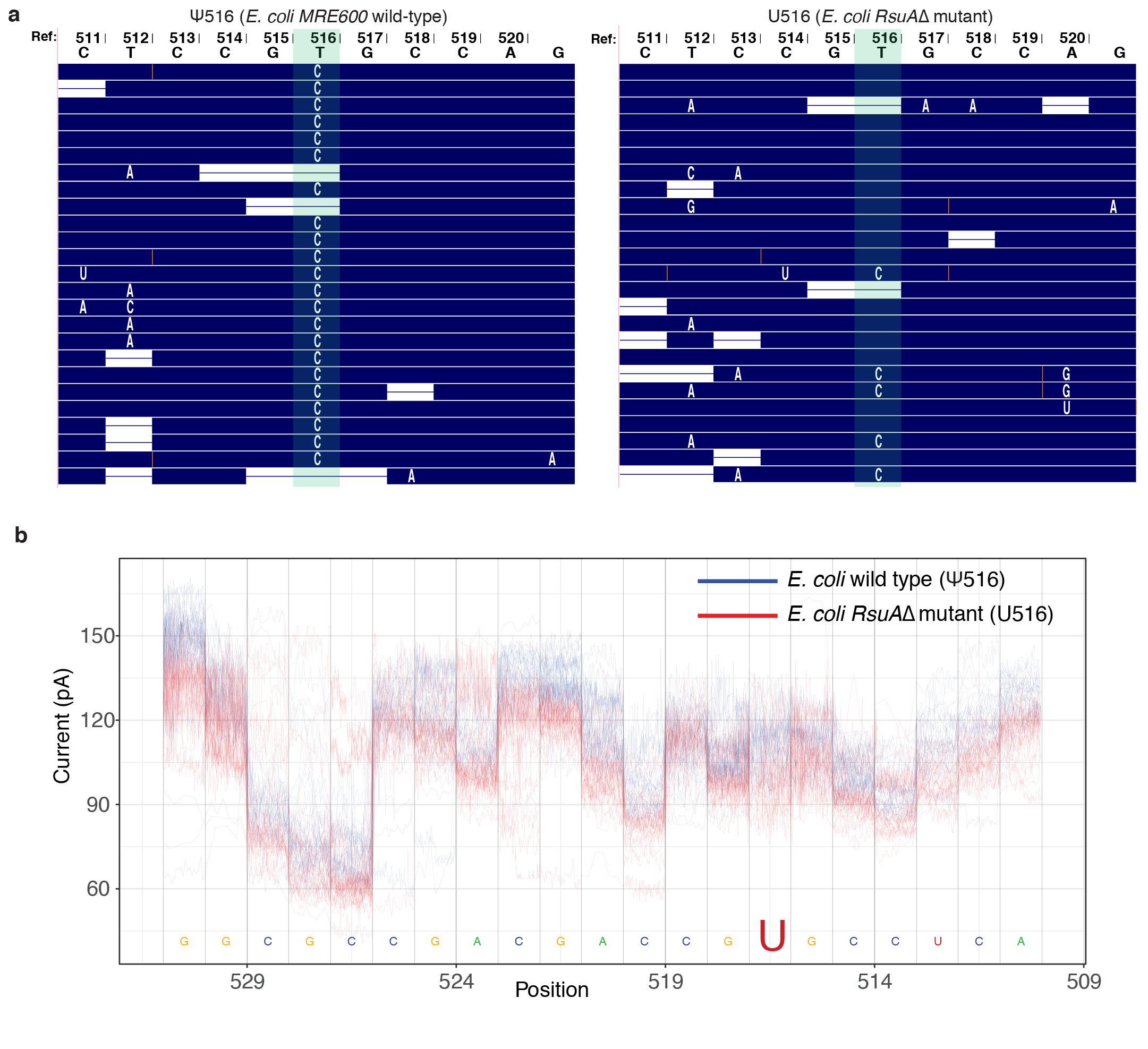

Supplement: S5 Fig — (a) Comparison of aligned reads from strands containing putative pseudouridine versus strands bearing canonical uridine at position 516. Reads are aligned to the E. coli MRE600 rrnD 16S rRNA reference sequence. Shown are twenty-five 16S rRNA reads from separate sequencing runs for E. coli str. MRE600 (wild type), which bears a pseudouridine at U516 (Ψ516) and an RsuA deficient strain (RsuAΔ mutant), which has a canonical U at position 516. Green shading indicates the position of U516 (shown as a T in the reference gene sequence). (b) Aligned ionic current traces from approximately thirty 16S rRNA reads covering position U516 from wild-type E. coli and RsuAΔ mutan strain. Pseudouridylation site, U516, is shown in large font. The sequence is shown 3′-to-5′ because ionic current signal is 3′-to-5′. Numbering uses standard E. coli 16S rRNA numbering. (PNG) [file pone.0216709.s005.png]
