# Supplementary material for: Reading canonical and modified nucleobases in 16S ribosomal RNA using nanopore native RNA sequencing
Source: PLoS One. 2019 May 16;14(5):e0216709. doi: 10.1371/journal.pone.0216709 (PMC6522004; doi:10.1371/journal.pone.0216709)
Supplement: S1 Table — Error models were estimated using marginAlign (guide alignments from BWA MEM “-x ont2d” followed by chaining). Statistics were generated using marginStats. (DOCX) [file pone.0216709.s006.docx]

**S1 Table.** Error rate profile for Enolase 2 transcript and 16S *E. coli* rRNA. Error models were estimated using marginAlign (guide alignments from BWA MEM “-x ont2d” followed by chaining). Statistics were generated using marginStats.

| **Median** | **RNA CS (Yeast Enolase 2; 1.35 kb)** | **16S *E. coli* rRNA (1.6 kb)** |
| --- | --- | --- |
| **Alignment identity** | 87.10% | 81.59% |
| **Insertions** | 2.69% | 1.90% |
| **Deletions** | 2.97% | 6.02% |
| **Mismatches** | 5.20% | 7.20% |
| **Read coverage** | 96.14% | 97.57% |
| **Read length** | 1256 bases | 1349 bases |
