# Supplementary material for: Reading canonical and modified nucleobases in 16S ribosomal RNA using nanopore native RNA sequencing
Source: PLoS One. 2019 May 16;14(5):e0216709. doi: 10.1371/journal.pone.0216709 (PMC6522004; doi:10.1371/journal.pone.0216709)
Supplement: S2 Table — Over and Under represented 5-mers comparison for Enolase 2 (left) and E. coli 16S rRNA (right). 5-mers were counted and compared for RNA read data and their respective reference sequences. LogFC represents log fold-change. (DOCX) [file pone.0216709.s007.docx]

**S2 Table.** Over and Under represented 5-mers comparison for Enolase 2 (left) and *E. coli* 16S rRNA (right)**.** 5-mers were counted and compared for RNA read data and their respective reference sequences. LogFC represents log fold-change.

| Reference | logFC | Read | logFC |  | Reference | logFC | Read | logFC |
| --- | --- | --- | --- | --- | --- | --- | --- | --- |
| AAAAA | -Inf | CCUAG | 1.016672501 |  | AAAAA | -Inf | CCCCC | 1.749887571 |
| AAAAU | -Inf | CUAGG | 1.016672501 |  | AAAAU | -Inf | GGGGG | 1.749887571 |
| AAAUA | -Inf | CUAAU | 0.884952046 |  | AAAUA | -Inf | GGGGU | 1.339474436 |
| AAUAA | -Inf | AUUAG | 0.884952046 |  | AAUAA | -Inf | ACCCC | 1.339474436 |
| AAUAU | -Inf | CUCUA | 0.74083179 |  | AAUAG | -Inf | CCCCU | 1.189478602 |
| AAUAG | -Inf | UAGAG | 0.74083179 |  | AAGAU | -Inf | AGGGG | 1.189478602 |
| AAUAC | -Inf | CGUAU | 0.723533092 |  | AUAUA | -Inf | CCUCC | 1.174672952 |
| AAUGC | -Inf | AUACG | 0.723533092 |  | AUAUG | -Inf | GGAGG | 1.174672952 |
| AACAG | -Inf | CACCC | 0.683502917 |  | AUAUC | -Inf | CCUAG | 1.148044234 |
| AACUA | -Inf | GGGUG | 0.683502917 |  | AUAGA | -Inf | CUAGG | 1.148044234 |
| AACUC | -Inf | CUAGA | 0.674894657 |  | AUAGU | -Inf | CCCUC | 1.121735811 |
| AACGA | -Inf | UCUAG | 0.674894657 |  | AUAGG | -Inf | GAGGG | 1.121735811 |
| AUAAA | -Inf | CGUAG | 0.672293688 |  | AUAGC | -Inf | UCGUA | 0.916374974 |
| AUAAU | -Inf | CUACG | 0.672293688 |  | AUUUA | -Inf | UACGA | 0.916374974 |
| AUAAG | -Inf | CUAGC | 0.625941895 |  | AUUUU | -Inf | CUCCC | 0.89152066 |
| AUAUA | -Inf | GCUAG | 0.625941895 |  | AUGUA | -Inf | GGGAG | 0.89152066 |
| AUAUU | -Inf | UAGCU | 0.621191129 |  | AUCUU | -Inf | GGGGA | 0.848227969 |
| AUAUG | -Inf | AGCUA | 0.621191129 |  | AGACG | -Inf | UCCCC | 0.848227969 |
| AUAUC | -Inf | CCCUU | 0.60666333 |  | AGGAC | -Inf | CCCUU | 0.775983801 |
| AUAGA | -Inf | AAGGG | 0.60666333 |  | ACAUA | -Inf | AAGGG | 0.775983801 |
