# Supplementary material for: Reading canonical and modified nucleobases in 16S ribosomal RNA using nanopore native RNA sequencing
Source: PLoS One. 2019 May 16;14(5):e0216709. doi: 10.1371/journal.pone.0216709 (PMC6522004; doi:10.1371/journal.pone.0216709)
Supplement: S3 Table — MRE600 reference sequence for rrnD 16S rRNA. (DOCX) [file pone.0216709.s008.docx]

**S3 Table.** Sequence variants in E. coli MRE600 16S rRNA MinION reads based on alignments to E. coli str. MRE600 reference sequence for rrnD 16S rRNA. Reference refers to the *E. coli* MRE600 16S rRNA gene from GenBank acquisition gb|CP014197.1|:343854-345409 (+) strand. miscalls within +/- 4 nt of a known modified nucleotide position are noted.

| Reference | Position | Reference nucleotide | Predicted SNV | Posterior probability | Type |
| --- | --- | --- | --- | --- | --- |
| ecoli_MRE600 | 6 | G | A | 0.323009035 | Likely miscall |
| ecoli_MRE600 | 79 | A | G | 0.477024303 | Documented base variant, 5 copies |
| ecoli_MRE600 | 90 | U | C | 0.495124202 | Documented base variant, 5 copies |
| ecoli_MRE600 | 183 | C | U | 0.334402802 | Documented base variant, 3 copies |
| ecoli_MRE600 | 226 | G | A | 0.455808427 | Likely miscall |
| ecoli_MRE600 | 273 | U | A | 0.304643919 | Documented base variant, 2 copies |
| ecoli_MRE600 | 288 | A | G | 0.38459441 | Likely miscall |
| ecoli_MRE600 | 328 | C | U | 0.340478205 | Likely miscall |
| ecoli_MRE600 | 346 | G | A | 0.301363107 | Likely miscall |
| ecoli_MRE600 | 485 | U | C | 0.760495972 | Likely miscall |
| ecoli_MRE600 | 516 | U | C | 0.908639215 | Known pseudouridine[[1]](https://paperpile.com/c/DXwBnL/2nrV) |
| ecoli_MRE600 | 527 | G | C | 0.778486859 | Known m7-guanosine[[1]](https://paperpile.com/c/DXwBnL/2nrV) |
| ecoli_MRE600 | 790 | A | G | 0.496515677 | Likely miscall |
| ecoli_MRE600 | 893 | C | U | 0.43893419 | Likely miscall |
| ecoli_MRE600 | 1150 | A | U | 0.512479558 | Likely miscall |
| ecoli_MRE600 | 1195 | C | U | 0.701573199 | Likely miscall |
| ecoli_MRE600 | 1281 | U | C | 0.702653464 | Documented base variant, 5 copies |
| ecoli_MRE600 | 1304 | G | A | 0.605698867 | Likely miscall |
| ecoli_MRE600 | 1380 | U | C | 0.40811185 | Likely miscall |
| ecoli_MRE600 | 1406 | U | C | 0.326475023 | Miscall, proximal to a modified nucleotide[[1]](https://paperpile.com/c/DXwBnL/2nrV) |
| ecoli_MRE600 | 1421 | G | A | 0.317460212 | Likely miscall |
| ecoli_MRE600 | 1495 | U | A | 0.568537937 | Proximal to a known 3-methyluridine |
| ecoli_MRE600 | 1518 | A | U | 0.390200124 | Known N6-dimethyladenosine[[1]](https://paperpile.com/c/DXwBnL/2nrV) |
| ecoli_MRE600 | 1519 | A | U | 0.467437353 | Known N6-dimethyladenosine[[1]](https://paperpile.com/c/DXwBnL/2nrV) |

References:

1. [Andersen NM, Douthwaite S. YebU is a m5C methyltransferase specific for 16 S rRNA nucleotide 1407. J Mol Biol. 2006;359: 777–786.](http://paperpile.com/b/DXwBnL/2nrV)
