# Supplementary material for: Reading canonical and modified nucleobases in 16S ribosomal RNA using nanopore native RNA sequencing
Source: PLoS One. 2019 May 16;14(5):e0216709. doi: 10.1371/journal.pone.0216709 (PMC6522004; doi:10.1371/journal.pone.0216709)
Supplement: S5 Table — MRE600 reference sequence for rrnD 16S rRNA. (DOCX) [file pone.0216709.s010.docx]

**S5 Table.** Sequence variants in E. coli *RsuA*Δ 16S rRNA MinION reads based on alignments to E. coli str. MRE600 reference sequence for rrnD 16S rRNA. Reference refers to the *E. coli* MRE600 16S rRNA gene from GenBank acquisition gb|CP014197.1|:343854-345409 (+) strand.

| Reference | Position | Reference nucleotide | Predicted SNV | Posterior probability |
| --- | --- | --- | --- | --- |
| ecoli_MRE600 | 226 | G | A | 0.490507026 |
| ecoli_MRE600 | 288 | A | G | 0.306708775 |
| ecoli_MRE600 | 485 | T | C | 0.576338051 |
| ecoli_MRE600 | 527 | G | C | 0.649247001 |
| ecoli_MRE600 | 790 | A | G | 0.342077249 |
| ecoli_MRE600 | 893 | C | T | 0.45459968 |
| ecoli_MRE600 | 1150 | A | T | 0.419609856 |
| ecoli_MRE600 | 1195 | C | T | 0.45034561 |
| ecoli_MRE600 | 1281 | T | C | 0.678837087 |
| ecoli_MRE600 | 1304 | G | A | 0.458438936 |
| ecoli_MRE600 | 1380 | T | C | 0.344505856 |
| ecoli_MRE600 | 1406 | T | C | 0.33249094 |
| ecoli_MRE600 | 1421 | G | A | 0.340091649 |
| ecoli_MRE600 | 1495 | T | A | 0.507183989 |
| ecoli_MRE600 | 1518 | A | C | 0.343415068 |
| ecoli_MRE600 | 1519 | A | T | 0.321498213 |
