# Supplementary material for: Reading canonical and modified nucleobases in 16S ribosomal RNA using nanopore native RNA sequencing
Source: PLoS One. 2019 May 16;14(5):e0216709. doi: 10.1371/journal.pone.0216709 (PMC6522004; doi:10.1371/journal.pone.0216709)
Supplement: S6 Table — (DOCX) [file pone.0216709.s011.docx]

**S6 Table.** Performance measurements for long 16S rRNA reads (>1000 bases) when classified against the SILVA database.

**Specificity**

| rRNA source organism | (Family name)  % | (Genus name)  % | (Species name)  % |
| --- | --- | --- | --- |
| E.coli str MRE600 | *(Enterobacteriaceae)*  96.79 | *(Escherichia)*  94.26 | *(Escherichia coli)*  83.89 |
| V.cholerae A1552 | *(Vibrionacea*e)  97.41 | *(Vibrio)*  94.50 | *(Vibrio cholerae)*  78.90 |
| M.maripaludis S2 | *(Methanococcaceae)*  97.75 | *(Methanococcus)*  94.93 | *(Methanococcus maripaludis)*  78.53 |
| S.enterica LT2 | *(Enterobacteriaceae)*  97.58 | *(Salmonella)*  97.10 | *(Salmonella enterica)*  88.59 |
| **Average** | **97.38** | **95.20** | **82.48** |

**Sensitivity**

| rRNA source organism | (Family name)  % | (Genus name)  % | (Species name)  % |
| --- | --- | --- | --- |
| *E.coli* str. MRE600 | *(Enterobacteriaceae)*  99.12 | *(Escherichia)*  97.97 | *(Escherichia coli)*  78.35 |
| *V.cholerae* str. A1552 | *(Vibrionacea*e)  97.31 | *(Vibrio)*  97.31 | *(Vibrio cholerae)*  93.21 |
| *M.maripaludis* str. S2 | *(Methanococcaceae)*  96.27 | *(Methanococcus)*  96.00 | *(Methanococcus maripaludis)*  94.38 |
| *S.enterica* str. LT2 | *(Enterobacteriaceae)*  96.79 | *(Salmonella)*  89.42 | *(Salmonella enterica)*  63.85 |
| **Average** | **97.37** | **95.17** | **82.45** |
