# Supplementary material for: Reading canonical and modified nucleobases in 16S ribosomal RNA using nanopore native RNA sequencing
Source: PLoS One. 2019 May 16;14(5):e0216709. doi: 10.1371/journal.pone.0216709 (PMC6522004; doi:10.1371/journal.pone.0216709)
Supplement: S7 Table — (DOCX) [file pone.0216709.s012.docx]

**S7 Table.** Performance measurements for long 16S rRNA reads (>1000 bases) when classified against the GRD database.

**Specificity**

| rRNA source organism | (Family name)  % | (Genus name)  % | (Species name)  % |
| --- | --- | --- | --- |
| E.coli str MRE600 | *(Enterobacteriaceae)*  98.54 | *(Escherichia)*  97.85 | *(Escherichia coli)*  97.59 |
| V.cholerae A1552 | *(Vibrionacea*e)  98.75 | *(Vibrio)*  92.69 | *(Vibrio cholerae)*  90.64 |
| M.maripaludis S2 | *(Methanococcaceae)*  98.58 | *(Methanococcus)*  92.53 | *(Methanococcus maripaludis)*  90.53 |
| S.enterica LT2 | *(Enterobacteriaceae)*  99.04 | *(Salmonella)*  93.67 | *(Salmonella enterica)*  91.62 |
| **Average** | **98.72** | **94.18** | **92.60** |

**Sensitivity**

| rRNA source organism | (Family name)  % | (Genus name)  % | (Species name)  % |
| --- | --- | --- | --- |
| *E.coli* str. MRE600 | *(Enterobacteriaceae)*  99.28 | *(Escherichia)*  83.41 | *(Escherichia coli)*  77.91 |
| *V.cholerae* str. A1552 | *(Vibrionacea*e)  98.65 | *(Vibrio)*  98.65 | *(Vibrio cholerae)*  98.44 |
| *M.maripaludis* str. S2 | *(Methanococcaceae)*  99.17 | *(Methanococcus)*  99.12 | *(Methanococcus maripaludis)*  98.75 |
| *S.enterica* str. LT2 | *(Enterobacteriaceae)*  97.77 | *(Salmonella)*  95.74 | *(Salmonella enterica)*  95.55 |
| **Average** | **98.72** | **94.23** | **92.66** |
